# Role of microRNAs in host defense against porcine reproductive and respiratory syndrome virus infection: a hidden front line

**DOI:** 10.3389/fimmu.2024.1376958

**Published:** 2024-03-25

**Authors:** Xuewei Huang, Weiye Liu

**Affiliations:** College of Veterinary Medicine, Qingdao Agricultural University, Qingdao, China

**Keywords:** porcine reproductive and respiratory syndrome virus, microRNAs, infection, innate immunity, viral genome

## Abstract

Porcine reproductive and respiratory syndrome virus (PRRSV) is one of the most globally devastating viruses threatening the swine industry worldwide. Substantial advancements have been achieved in recent years towards comprehending the pathogenesis of PRRSV infection and the host response, involving both innate and adaptive immune responses. Not only a multitude of host proteins actively participate in intricate interactions with viral proteins, but microRNAs (miRNAs) also play a pivotal role in the host response to PRRSV infection. If a PRRSV–host interaction at the protein level is conceptualized as the front line of the battle between pathogens and host cells, then their fight at the RNA level resembles the hidden front line. miRNAs are endogenous small non-coding RNAs of approximately 20–25 nucleotides (nt) that primarily regulate the degradation or translation inhibition of target genes by binding to the 3’-untranslated regions (UTRs). Insights into the roles played by viral proteins and miRNAs in the host response can enhance our comprehensive understanding of the pathogenesis of PRRSV infection. The intricate interplay between viral proteins and cellular targets during PRRSV infection has been extensively explored. This review predominantly centers on the contemporary understanding of the host response to PRRSV infection at the RNA level, in particular, focusing on the twenty-six miRNAs that affect viral replication and the innate immune response.

## 1 Introduction

Porcine reproductive and respiratory syndrome (PRRS) is a high fever, high mortality, respiratory tract distress and reproductive failures in pregnant sows disease caused by PRRS virus (PRRSV) (1). Since the 1980s outbreak, PRRS has indisputably evolved into a huge financial issue, profoundly impacting pig production and inflicting catastrophic economic repercussions to the global swine industry (2). Currently, the economic losses attributable to PRRSV in the USA is estimated at nearly $600 million annually (2). The latest economic appraisal revealed that, in the worst-case scenario, the losses on farm profits in Germany attributed to PRRS are approximately -41% (3). As a worldwide swine pathogen that has led to substantial economic losses, PRRSV continues to be a subject of enduring and widespread concern (4).

PRRSV, an enveloped RNA virus with a diminutive 55 nm diameter, is classified within the *Arteriviridae* family, belonging to the *Nidovirales* order (5). The virus has two genotypes, namely type 1 and type 2, exhibiting a sequence identity between them of merely 50–60% and distinctive serotypic characteristics (6, 7). PRRSV strains exhibit extensive genetic and antigenic diversity, and frequently undergoing recombination processes that give rise to the emergence of various novel strains (8, 9). As an illustration, the highly pathogenic PRRSV (HP-PRRSV) strain established endemicity following an abrupt outbreak in China in 2006 (10). Despite the modified live vaccine (MLV) has been widely used, and various MLVs targeting both genotypes have been formulated, the continued emergence of novel virulent PRRSV caused global outbreaks, posing an ongoing threat to the swine industry (11–13). More effective PRRSV-specific treatment or vaccines are and will be in great demand for the control of PRRS. Therefore, gaining a comprehensive insight into the pathogenesis of PRRSV infection will significantly contribute to the development of novel vaccines.

MicroRNAs (miRNAs) are a class of non-coding RNAs, that regulate gene expression by targeting the 3′ UTR of specific mRNAs. The biogenesis of miRNAs have been well elucidated (14). In brief, miRNAs are initially transcribed as primary transcripts (pri-miRNA). Pri-miRNA undergo subsequent processing into approximately 70 nt precursor miRNA (pre-miRNA) in the nucleus by the RNase III nuclease Drosha complex. The pre-miRNA is then transported to the cytoplasm *via* exportin 5, where it undergoes cleavage, resulting in the generation of an approximately 22 nt mature miRNA duplex. In general, one strand (mature miRNA) is incorporated into the RNA-induced silencing complex (RISC) through associating with Argonaute (Ago) proteins, while the complementary strand undergoes degradation. Mature miRNAs, within the RISC, induce translational repression and deadenylation of target mRNA. Numerous host miRNAs have demonstrated influence on various processes, including pathogenic diseases and host immunity (4, 15, 16). Therefore, understanding the biological functions of miRNAs and the mechanism of their actions will be instrumental in drug development and disease control. The effect of miRNAs can be either supportive of the virus (proviral) or antiviral (17). On one hand, certain host miRNAs may function as antiviral factors suppressing PRRSV replication by directly targeting the PRRSV genome or host factors. For instance, miR-10a, miR-122, miR-218, ssc-miR-124a, miR-181, miR-506, miRNA let-7f-5p, miR-142-3p, miR-125b, miR-26a, miR-150, miR-331-3p, miR-210, miRNA let-7 family, miR-23, and miR-130b. On the other hand, viruses may exploit the host miRNAs to boost PRRSV replication. For instance, miR-376b-3p, miR-373, miR-30c, miR-541-3p, miR-382-5p, miR-142-5p, miR-29, miR-296-3p, miR-22 and miR-24-3p. Host miRNAs appear to function as either antiviral defenses or can be exploited by viruses to promote their proliferation. In this review, we encapsulate recent discoveries that specifically delve into the roles of miRNAs and underlying mechanisms involved in PRRSV infection. The insights offer a fresh perspective for comprehending the pathogenesis of PRRSV infection.

## 2 Virus characteristics

The PRRSV consists of a single-stranded, positive-sense RNA. It is comprised of 11 open reading frames (ORFs) and encodes a minimum of 16 non-structural proteins (Nsps), along with four membrane-associated glycoproteins (2-5) and two non-glycosylated membrane proteins (18–21). Upon PRRSV infection, the host cells recognize virus through pattern recognition receptors (PRRs). Subsequently, host cells mobilize essential resources to impede virus proliferation, whereas the invader, PRRSV, utilizes viral proteins to fight against the host response. Consequently, an ongoing life-and-death conflict persists between PRRSV and the host.

## 3 The battle between PRRSV and host

The struggle between pathogens and host persists incessantly. On the one hand, viruses depend on the host environment to complete their replication cycle, affecting the synthesis and metabolism of host cells. On the other hand, in response to the pathogen, the host also initiates complex antiviral immune responses to counteract the infectious agent. During PRRSV infection, the virus adeptly navigates the intricacies of the host immune system, demonstrating a remarkable capacity to evade host antiviral responses from both the innate and adaptive immune systems (17, 22). Furthermore, host can block the entry of PRRSV into cells through cellular receptors or factors (4).

### 3.1 Escape from host innate immune response

The innate immune response serves as the first line of host defense, restricting the viral spread and concurrently playing a pivotal role in initiating the adaptive immune response. Viruses are recognized by PRRs, initiating the adaptive immune response. (23, 24). Once activated, IRF3/7 and NF-κB translocate to the nucleus, where they prompt the transcription of type I interferon (IFN-I) and proinflammatory cytokines. The JAK–STAT signaling pathway can be initiated by IFN-I, leading to the transcription of interferon stimulated genes (ISGs). However, PRRSV has developed sophisticated strategies to impede the signaling cascade of the innate antiviral immunity. Several Nsps and structural proteins of PRRSV can selectively target different steps in this process, such as evading PRRs recognition, targeting the adaptors and kinase, disrupting transcription factors, or targeting of the JAK–STAT signaling pathway and ISGs (24, 25). This orchestrated evasion facilitates PRRSV in circumventing the host immune system, thereby promoting their own replication.

#### 3.1.1 Targeting the IFN-producing signaling pathway

In the early stages of infection, PRRSV inhibits IRF3 activation and IFN-β production by disrupting the activation of IPS-1 (26). PRRSV-2 Nsp1α, acting as a viral antagonist for IFNs, blocks the activation of NF-κB and production of IFN-β by inhibiting IκB phosphorylation (27). Moreover, Nsp1α is crucial for inhibiting IFNs by facilitating the degradation of CREB binding protein (CBP) to prevent the recruitment of CBP for the assembly of the enhanceosome, which is likely a pivotal mechanism in suppressing IFNs (28). Nsp1β, Nsp2, Nsp4, Nsp11, and N protein inhibits IFN-β induction by blocking the phosphorylation and nuclear translocation of the IRF3 or NF-κB (29–33).

#### 3.1.2 Targeting the JAK-STAT signaling pathway

A characteristic manifestation of PRRSV infection in swine involves a delay in the production of virus-neutralizing antibodies, which may be correlated with potential interference in JAK-STAT signaling mediated by cytokines (34). Studies have demonstrated that PRRSV employs various mechanisms to counteract the JAK-STAT signaling pathway. PRRSV was indicated to suppress JAK–STAT signaling *via* impeding the nuclear translocation of IFN-stimulated gene factor 3 (ISGF3), thereby inhibiting ISGs production (35, 36). Further investigations revealed that PRRSV Nsp 1β blocks ISGF3 nuclear translocation by inducing karyopherin-α1 (KPNA1) degradation, and that the inhibition is notably associated with Nsp1β valine-19 (37). Nsp11 antagonizes the interferon signaling through promoting the degradation of STAT2 or targeting IRF9 (36, 38). Additionally, PRRSV Nsp5 antagonize the JAK–STAT3 signaling by blocking STAT3 degradation (39). Additionally, PRRSV also moderate the antiviral immunity through manipulating ISGs expression (40–42).

### 3.2 Escape from adaptive immunity

PRRSV can also directly target adaptive immunity for evasion. Dendritic cells (DCs), recognized as formidable antigen-presenting cells with the ability to directly activate naive T cells, play a pivotal role in immune response initiation. Research findings indicate that PRRSV-1 infection did not induce the maturation of DC, thus impairing their typical antigen presentation ability (43). Previous studies demonstrated that PRRSV Nsp1α/4/2TF can individually reduce the expression of leukocyte antigen class I (SLA-I) on the cell surface, consequently disrupting SLA-I antigen presentation pathway (44–46).

### 3.3 PRRSV entry blockers

PRRSV exhibits a highly narrow cell tropism. Numerous studies have elucidated that PRRSV infection relies on cellular factors, including heparin sulfate (HS), porcine sialoadhesin, vimentin, CD151/163/169/209, and non-muscle myosin heavy chain 9 (MYH9) (6, 47). Particularly noteworthy is the pivotal role played by the SRCR5 domain of CD163 in PRRSV infection. Truncated forms of CD163 and CD169 have been shown to effectively inhibit PRRSV replication (48, 49). Moreover, blebbistatin also can impedes the replication of PRRSV (50).

## 4 Roles of miRNAs in host response to PRRSV infection

**Figure 1:**
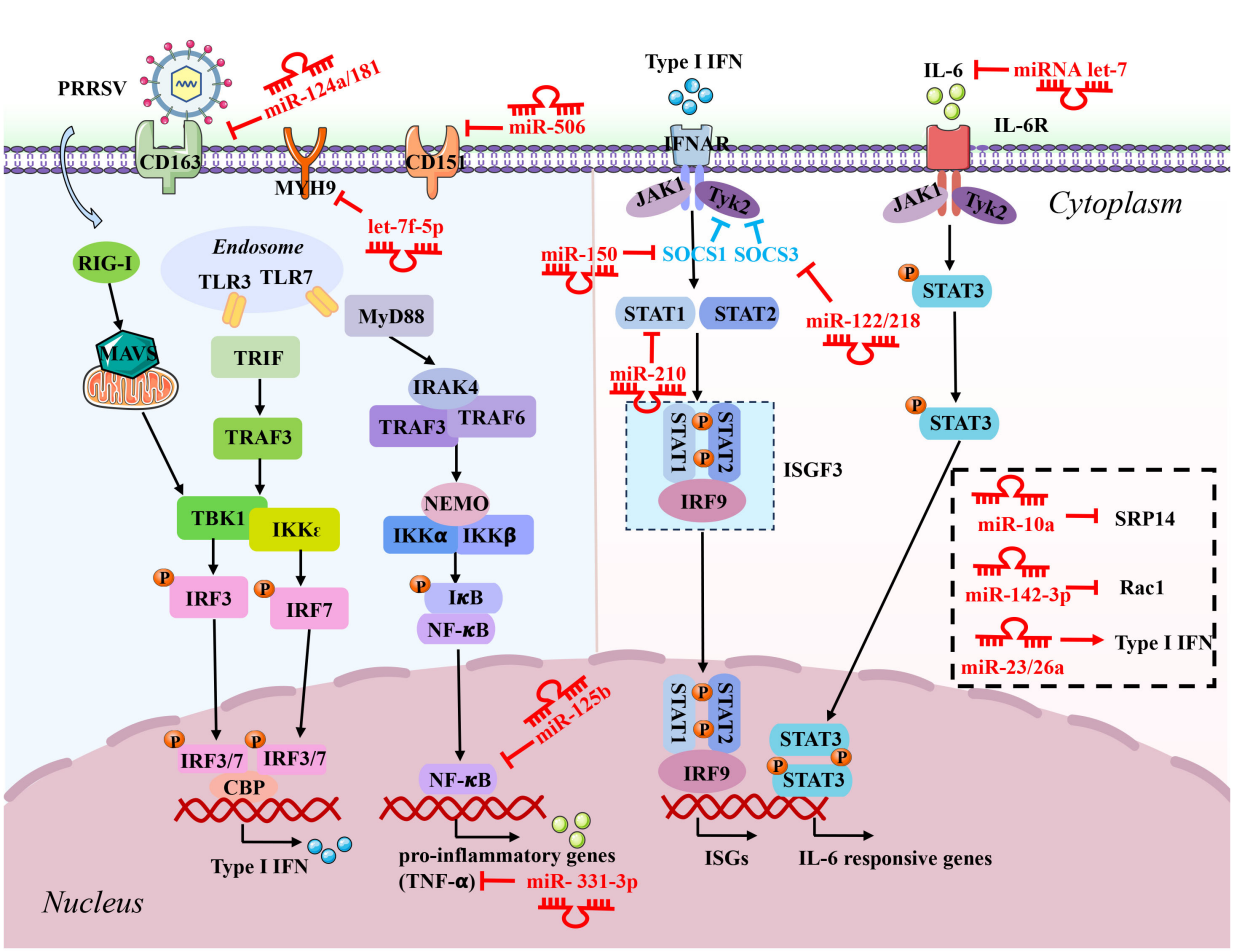
Schematic diagram of the roles of microRNAs (miRNAs) in host response to PRRSV infection. These miRNAs inhibit PRRSV replication by directly targeting the negative regulators involved in the immune signaling pathways, including interferons and pro-inflammatory genes producing signaling pathways and IFN-JAK/STAT-ISGs pathways. The red inhibitory arrow indicates that the host factors directly targeted by miRNAs. P, phosphate.

**Figure 2:**
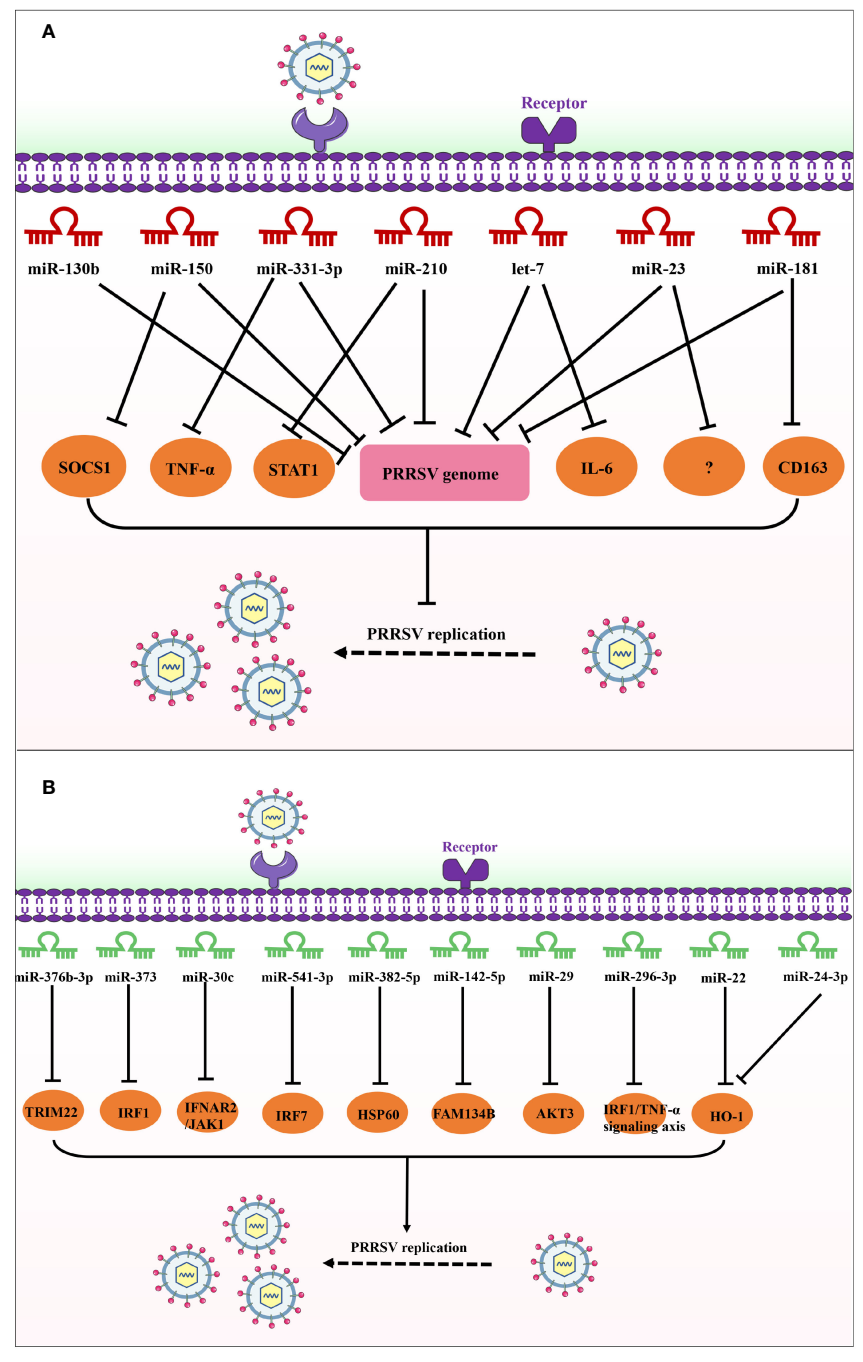
Schematic diagram of the roles of miRNAs in host response to PRRSV infection. **(A)** These miRNAs inhibit PRRSV replication by directly targeting the viral genome; **(B)** These miRNAs promote PRRSV replication by targeting antiviral host factors or signaling pathways.

miRNAs can regulate diverse cellular processes by directly binding to mRNA, thereby manipulating protein levels (14). Evidence is accumulating that miRNAs can impact virus replication through binding to the virus genome or host factors in antiviral-related signaling pathways (15). Recently, twenty-four miRNAs have been reported to participate in modulating PRRSV replication. Based on their effects on PRRSV infection, miRNAs can be categorized into two groups: (i) those that promote PRRSV replication and (ii) those that inhibit virus replication. Group one includes sixteen miRNAs (miR-10a, miR-122, miR-218, ssc-miR-124a, miR-181, miR-506, miRNA let-7f-5p, miR-142-3p, miR-125b, miR-26a, miR-150, miR-331-3p, miR-210, miRNA let-7 family, miR-23, and miR-130b) exerting antiviral effects on PRRSV infection, while group two is composed of ten miRNAs (miR-376b-3p, miR-373, miR-30c, miR-541-3p, miR-382-5p, miR-142-5p, miR-29, miR-296-3p, miR-22 and miR-24-3p) facilitating PRRSV replication by suppressing host defense. The details of miRNAs targeting host factors or PRRSV genome are illustrated in **Figures 1**, **2**, **Table 1**.

**Table 1:** Summary of miRNAs studies in PRRSV infection.

miRNA	miRNA expression	Targets or signaling pathways	Functions
miR-10a	up-regulation	SRP14	Inhibit PRRSV replication
miR-122	down-regulation	SOCS3	
miR-218	down-regulation	SOCS3	
ssc-miR-124a	down-regulation	CD163	
miR-181	up-regulation	CD163/PRRSV ORF4	
miR-506	/	CD151	
let-7f-5p	down-regulation	MYH9	
miR-142-3p	down-regulation	Rac1	
miR-125b	/	NF-κB signaling pathway	
miR-26a	/	Activating the IFN-I pathway	
miR-150	up-regulation	PRRSV genome/SOCS1	
miR-331-3p	up-regulation	PRRSV-2 ORF1b/TNF-α	
miR-210	up-regulation	PRRSV-2 ORF1b/STAT1	
let-7 family	up-regulation	PRRSV-2 genome/IL-6	
miR-23	up-regulation	PRRSV-2 genome/ Activating IRF3/IRF7	
miR-130b	/	PRRSV genome	
miR-376b-3p	up-regulation	TRIM22	promote PRRSV replication
miR-373	up-regulation	IRAK1/IRAK4/NFIA/NFIB/IRF1	
miR-30c	up-regulation	IFNAR2/JAK1	
miR-541-3p	up-regulation	IRF7	
miR-382-5p	up-regulation	HSP60	
miR-142-5p	up-regulation	FAM134B	
miR-29	up-regulation	AKT3	
miR-296-3p	down-regulation	Attenuating IRF1/TNF-α signaling axis	
miR-22	/	HO-1	
miR-24-3p	up-regulation	HO-1	

### 4.1 miRNAs inhibiting PRRSV infection

miRNAs play a crucial role in combating PRRSV infection thought directly targeting of the negative regulators of the immune signaling pathways to boost the innate immunity or by targeting the viral genome to impede viral replication. Up to now, sixteen miRNAs (miR-10a, miR-122, miR-218, ssc-miR-124a, miR-181, miR-506, miRNA let-7f-5p, miR-142-3p, miR-125b, miR-26a, miR-150, miR-331-3p, miR-210, miRNA let-7 family, miR-23, and miR-130b) have been found to inhibit PRRSV infection.

#### 4.1.1 miRNAs targeting host factors involved in PRRSV replication

miR-10a belonging to the miR-10 family, is encoded by pig chromosome 12 and is participated in immune-related responses and cancer. Interestingly, the expression of miR-10a is evidently upregulated during PRRSV infection, and further inhibited PRRSV replication through targeting signal-recognition particle 14 (SRP14) (**Figure 1**, **Table 1**). SRP14 plays a role in the synthesis of the PRRSV genome through its interaction with Nsp (51). Additionally, additional investigations revealed that IRF8 serves as a negative regulator of miR-10a. PRRSV infection decreases the expression level of IRF8, resulting in the upregulation of miR-10a and thereby exerting an anti-PRRSV role (52). In addition to PRRSV, there are a few reports about the antiviral effects of miR-10a on other viruses. Previous studies demonstrated that miR-10a-5p inhibits porcine hemagglutinating encephalomyelitis virus (PHEV) replication thought directly targets the Syndecan 1 (SDC1), and exerting an antiviral effect (53). Conversely, miR-10a upregulates the expression of white spot syndrome virus (WSSV) *via* targeting its 5’ untranslated region (UTR) (54). Thus, it appears that miR-10a plays a dual role in viral infections, serving as a double-edged sword by exhibiting both antiviral effects and promoting viral replication.

miR-122 and miR-218 located on pig chromosome 1 and chromosome 8, respectively. They involve multiple roles including cancers development and antiviral responses (55–58). It was reported that overexpression of miR-122 or miR-218 significantly inhibits PRRSV replication thought targeting the 3’ UTR region of porcine suppressor of cytokine signaling 3 (SOCS3) in Marc-145 cells (**Figure 1**, **Table 1**) (59, 60). SOCS3 is recognized as a negative regulator of JAK-STAT signaling (61). The modulation of SOCS3 expression by miR-122/218 facilitate the expression of IFN-β and ISGs, subsequently playing an important role in the host antiviral response. However, unlike miR-10a, the expression level of miR-122/218 are significantly decreased during PRRSV infection, indicating that miR-122/218 may hardly exert an antiviral activity because PRRSV infection inhibits the expression of miR-122/218. miR-122 also can facilitate rhinoviruses-induced lung disease *via* targeting the SOCS1 (62). Besides, miR-122 may has different effects on other viruses. For example, miR-122 can promote hepatitis C virus (HCV) proliferation by PLK1-ELAVL1/HuR-miR-122 signaling (63), and anti-miR-122 oligonucleotides has been successfully trialed for treatment of HCV (64), which provides a solid foundation for employing cellular miRNAs in the viral infections. It is noteworthy that miR-218 can function as a tumor suppressor in cancers. Liu et al. (56) reported that miR-218 inhibited tumor cell migration by targeting roundabout guidance receptor 1 (ROBO1) in cervical cancer.

Several miRNAs are able to target the cellular receptor of PRRSV, such as ssc-miR-124a, miR-181, miR-506, and miRNA let-7f-5p. It was reported that ssc-miR-124a and miR-181 inhibits PRRSV replication by directly targeting CD163 receptor (**Figure 1**, **Table 1**) (65, 66), and miR-506 and miRNA let-7f-5p can inhibit PRRSV replication *via* directly targeting CD151 and MYH9, respectively (**Figure 1**, **Table 1**) (67, 68). Of note, CD163, CD151, and MYH9 serve as a crucial cellular receptor for PRRSV, playing an indispensable role in mediating the entry of PRRSV into host cells (49, 69, 70). Interestingly, previous study has demonstrated that miR-181 is upregulated in PRRSV-infected blood monocytes and PAMs, and delivered miR-181 mimics can strongly inhibit PRRSV replication through targeting PRRSV ORF4 (**Figure 2**, **Table 1**) (71). Additionally, the investigation indicated the expression levels of miR-181 is upregulated in the spleen, muscle, and alveolar macrophages of pigs during PRRSV infection, and target gene prediction further indicated that miR-181 exhibits perfect complementarity with Foxp1, Ddx3x, Nfat5, and Mpp5 (72). However, a deeper understanding of miR-181 and its target gene requires further exploration. A recent publication showed that miR-181 also can remarkably inhibit the HIV-1 viral protein P24 via targeting DDX3X (73). Notably, similar to the miRNAs as miR-122/218, the expression level of miR-124a and let-7f-5p are observably downregulated during PRRSV infection, suggesting that miR-124a and let-7f-5p may hardly exert an antiviral activity. Apart from PRRSV, miR-124a has been extensively studied in lung cancer, where it acts as a tumor suppressor by targeting STAT3, AKT-GLUT1/HKII, and Rab32 (74, 75). Therefore, it is evident that miR-124a functions not only as a tumor suppressor but also as an antiviral factor. Currently, there is no existing report on the impact of let-7f-5p on other viral infections or cancers. Interestingly, miR-506 has been identified to play a crucial role in various types of cancer, such as breast cancer, colon cancer, and cervical cancer, etc. (76). Simultaneously, additional efforts are required to validate the potential value of miR-506 as a diagnostic biomarker.

miRNAs may exert additional effects to prevent PRRSV function. For example, miR-142-3p and miR-125b inhibit PRRSV replication by directly targeting Ras-related C3 botulinum toxin substrate 1 (Rac1) and negatively regulating NF-κB signaling pathway, respectively (**Figure 1**, **Table 1**) (77, 78). Moreover, miR-26a can impede PRRSV replication by activating the IFN-I pathway and promoting the ISGs production (79). Nevertheless, specific targets for the miRNAs of both miR-125b and miR-26a within these signaling pathways remain unidentified. Presently, miR-142-3p, miR-125b, and miR-26a also has been extensively investigated and confirmed as key regulators in diverse cancers (80–82), suggesting that these miRNAs play multifaceted roles in biological processes.

#### 4.1.2 miRNAs targeting the PRRSV genome

It has been documented that host miRNAs can target RNA viral genomes, representing a newly discovered host antiviral defense mechanism. As described above, miR-181 suppresses PRRSV proliferation by targeting PRRSV ORF4 and PRRSV receptor CD163 (**Figure 2A**, **Table 1**) (65). The expression of miR-150 is observably upregulated after PRRSV infection, and miR-150 suppresses PRRSV replication via directly targeting both the PRRSV genome and SOCS1 (**Figure 2A**, **Table 1**) (83). Moreover, previous studies have highlighted the pivotal role of miR-150-5p in SARS-CoV-2 infection, demonstrating its ability to inhibit viral infection by directly interacting with Nsp10 (84).

The expression of miR-331-3p and miR-210 is markedly increased in PRRSV-2 infected PAMs. Similar to miR-150, miR-331-3p and miR-210 inhibit PRRSV replication by two mechanisms. On one hand, they directly targeting PRRSV-2 ORF1b (85). Of note, PRRSV ORF1b plays a significant role in viral pathogenicity and virulence (86), and disrupting ORF1b significantly impacts PRRSV replication (87). On the other hand, miR-331-3p and miR-210 inhibit the expression of porcine TNF-α/STAT1 by targeting their 3′UTR, thereby suppressing PRRSV replication (**Figure 2A**, **Table 1**). It was reported that TNF-α plays a key role in lung injury during HP-PRRSV-infection (87), and STAT1 can upregulated TNF-α expression. Importantly, the study indicated that intramuscular injection of the expression plasmid of miR-331-3p can attenuate lung injury and markedly inhibit PRRSV replication *in vivo*, which provides a prosperous basis for the treatment of PRRSV infection using cellular miRNAs. Of note, an accumulating body of evidence indicated that miR-331-3p is a tumor-suppressor miRNA in various cancers by targeting multiple oncogenes (88–90).

The let-7 family of miRNAs was initially recognized in C. elegans as a pivotal gene participating in embryonic development (91). Accumulating evidence has confirmed that let-7 plays a key role in regulating immune responses (92, 93). Upon PRRSV infection, the expression of let-7 family is significantly upregulated, and the let-7 family inhibits PRRSV proliferation via directly targeting both the PRRSV-2 genome and IL-6 (**Figure 2A**, **Table 1**) (16). More importantly, let-7 family can remarkably decrease PRRSV infection and associated pathological changes both *in vitro* and *in vivo*, suggesting that the let-7 family may serve as promising therapeutic targets for PRRS.

The let-7 family has garnered significant attention, and numerous scholarly articles have delved into exploring the intricate roles of let-7 in the host response to viral infections (93). Letafati et al. reported that (94) miR-let-7c can significantly inhibit HCV replication by stimulating the expression of heme oxygenase-1 (HO-1). Previous studies indicated that let-7b and let-7c can inhibit the replication of the HCV and influenza virus by directly targeting of inhibitors of IFN-I signaling and H1N1 M1 gene (95, 96), respectively. Additionally, let-7 family members (including let-7b, c, d, e, f, g, i) and miR-98 could inhibit COVID-19 by targeting the S protein and M protein (97). Therefore, the let-7 family exhibits promising potential as a therapeutic agent for viral diseases.

Likewise, miR-23 exerts multifaceted inhibitory impacts on PRRSV replication. miR-23 not only represses the PRRSV replication *via* targeting the PRRSV genome, but also enhance IFN-I induction through the activation of IRF3/IRF7 (**Figure 2A**, **Table 1**) (98). However, the expression of miR-23 and its specific targets within the pathways remain unclear. In the event of an elevation in miR-23 expression during PRRSV infection, it is conceivable that PRRSV employs certain mechanisms to enhance miR-23 expression for its survival. Conversely, in cases where miR-23 expression is down-regulated during PRRSV infection, the innate response against PRRSV infection should be enhanced.

miR-130b can exert inhibitory effects on PRRSV infection *via* directly targeting the viral genome, and intranasal inoculation of miR-130b provides partial protection against the lethal challenge posed by HP-PRRSV strains vJX143 (**Figure 2A**, **Table 1**) (99), which offers a crucial foundation for employing cellular miRNAs in the treatment of PRRSV infection. Apart from PRRSV, there are a few reports regarding the antiviral impact of miR-130b on other viruses. Fu et al. (100) reported that gga-miR-130b-3p inhibits IBDV replication by directly targeting both IBDV segment A and SOCS5 in DF-1 cells. gga-miR-130b-3p hampers the cell cycles by suppressing the expression of matrix metallopeptidase 2 (MMP2) and MMP9, which are intricately associated with cell invasion (101). Hence, it appears that miR-130b serves not only as an antiviral factor but also as a tumor suppressor.

### 4.2 miRNAs promoting PRRSV replication

PRRSV can exploit host miRNAs to manipulate host immunity in ways that facilitate viral replication. Up to now, ten pig miRNAs (miR-376b-3p, miR-373, miR-30c, miR-541-3p, miR-382-5p, miR-142-5p, miR-29, miR−296−3p, miR-22 and miR-24-3p) have been characterized to promote the proliferation of PRRSV by targeting antiviral host factors or signaling pathways.

It was reported that the expression of miR-376b-3p is remarkably increased in PRRSV-infected MARC-145 cells, and this upregulation facilitated the PRRSV replication and inhibited the tripartite motif-containing 22 (TRIM22) expression *via* targeting its 3’ UTR, impairing TRIM22-mediated anti-PRRSV activity (**Figure 2B**, **Table 1**) (102). TRIM22 as a key restriction can inhibit PRRSV replication thought interacting with the N protein (103). However, there is no available report concerning the impact of miR-376b-3p on host defense against other viruses in the present study.

In analogous investigations, the expression of miR-373 is markedly upregulated in PRRSV-infected cells, and negatively regulates the production of IFN-β by directly targeting IRAK1/4, NFIA/B, and IRF1 (**Figure 2B**, **Table 1**) (104). NFIA and NFIB, identified as novel proteins inducing IFN-β production, demonstrate inhibitory effects on PRRSV replication. Moreover, miR-373 also plays a crucial role in other virus infection. For example, Mukherjee et al. (105) reported that miR-373 is upregulated and targeted JAK1 and IRF9 during HCV infection, thereby attenuating the IFN-I signaling pathway and promoting HCV replication. Gong et al. (106) reported that miR-373 promotes HCV replication by activating of IFN-I responses through directly targeting IRF5 in host cells. More efforts will be necessary to elucidate the role of miR-373 in the host response to viral infections.

The miR-30c plays a crucial role in multiple pathogenic infections. Reportedly, the expression of miR-30c is significantly upregulated in PRRSV-infected host cells. miR-30c is found to impair the IFN-I signaling by targeting the 3’ UTR of IFNAR2 and JAK1, ultimately promoting the replication of PRRSV (**Figure 2B**, **Table 1**) (107, 108). In addition to PRRSV, gga-miR-30c-5p also can facilitated the replication of fowl adenovirus serotype 4 by directly targeting myeloid cell leukemia-1 (109). Avian reovirus (ARV) significantly upregulates the gga-miR-30c-5p expression, and gga-miR-30c-5p can inhibit the ARV-induced autophagy by targeting autophagy related 5, thereby suppressing ARV proliferation (110). Wang et al. (111) reported that overexpression of miR-30c-5p suppresses porcine epidemic diarrhea virus (PEDV) infection through targeting SOCS1. However, the expression of miR-30c-5p is downregulated during PEDV infection. Therefore, PEDV can escape antiviral immune responses by engaging miR-30c-SOCS1 axis. As described above, the miR-30c family exhibits dual roles, acting either as a proviral or antiviral factor, in the host response to viral infections.

PRRSV-2 infection can upregulate the expression of miR-541-3p and miR-382-5p in MARC-145 cells, respectively. miR-541-3p and miR-382-5p facilitated PRRSV-2 replication by reducing IFN-I production and directly targeting IRF7 and heat shock protein 60 (HSP60), respectively (**Figure 2B**, **Table 1**) (112, 113). More importantly, IRF7 and HSP60 are antiviral protein against PRRSV replication. Additionally, Guan et al. (114) reported that PRRSV-induces miR-142-5p facilitated PRRSV replication through directly targeting FAM134B (**Figure 2B**, **Table 1**). Of note, PRRSV replication occurs within the endoplasmic reticulum (ER), and the activation of the IFN-I signaling pathway can be facilitated through FAM134B-mediated ER-phagy. Furthermore, it was confirmed that the expression of miR-29a markedly increases after PRRSV infection, promoting PRRSV replication *via* targeting AKT3 3’UTR (**Figure 2B**, **Table 1**) (115). Similarly, new data indicated that overexpression of miR-296-3p promotes HP-PRRSV replication by attenuating IRF1/TNF-α signaling axis (**Figure 2B**, **Table 1**) (116). However, unlike miR-541-3p, miR-382-5p, and miR-142-5p, the expression level of miR-296-3p is remarkably downregulated after PRRSV infection, indicating that the host may enhance antiviral responses by suppressing miR-296-3p expression. However, the roles of miR-541-3p, miR-382-5p, miR-142-5p, miR-29a, and miR-296-3p in other viral infections have not been reported.

The miR-22 gene plays a critical role in the progression and metastases of multiple cancer cells through multiple processes and acts as both an oncomiR and a tumor-suppressor (117, 118). Besides, miR-24-3p can regulate the epithelial–mesenchymal transition (EMT) process in retinoblastoma and lung cancer (119, 120) and inhibit the progression of pancreatic ductal adenocarcinoma (121). Notably, the abnormal expression of miR-22-5p is found in PBMC of crossbred pigs following treatment with the classical swine fever vaccine virus, which suggesting that miR-22-5p may play a key role in virus infection (122). It was reported that miR-22 and miR-24-3p promote PRRSV proliferation through suppressing the expression of HO-1 (**Figure 2B**, **Table 1**) (123, 124).

## 5 A therapeutic strategy targeting PRRSV through the RNA silencing pathway

RNA silencing serves as a natural antiviral defense in mammals (125). In addition to host miRNAs, various RNA silencing pathways, such as small interfering RNAs (siRNAs), artificial miRNAs, and short-hairpin RNAs (shRNAs) have been systematically examined for their inhibitory potential against PRRSV infection, both *in vitro* and *in vivo*. In the battle against PRRSV invasion, hosts employ not only endogenous miRNAs but also siRNAs, shRNAs, and artificial miRNAs to modulate PRRSV replication. Notably, siRNAs have been reported to impede PRRSV replication in permissive cell lines by targeting Nsp1α/9 and N genes of PRRSV (126–128). Moreover, Bao et al. (129) demonstrated that four specific siRNAs can effectively inhibit the expression of the genes ORF1b/5/6/7 in cells, thereby suppressing the replication of PRRSV-JXwn06. Furthermore, Li et al. (130) indicated that two shRNAs targeting ORF1 region can observably inhibit PRRSV replication. It is worth noting that intranasal inoculation of piglets with either miR-130b mimics or miR-181 mimics provided protection against the lethal challenge posed by HP-PRRSV *in vivo* (72, 99). This finding lays a crucial foundation for the potential therapeutic use of miRNAs in treating PRRSV infections. Additionally, recombinant pseudorabies virus (PRV)-mediated siRNAs targeting the ORF7 effectively inhibited the replication of HP-PRRSV strain HN1 *in vitro* and mitigated gross lung lesions in piglets *in vivo* (131), highlighting siRNA mediated by recombinant PRV emerges as a promising and innovative approach for preventing HP-PRRSV infections in swine. In an effort to confer resistance against PRRSV, an RNA interference (RNAi) approach has been employed to generate transgenic (TG) pigs expressing PRRSV-specific shRNA. However, stabled expression of shRNA targeting the PRRSV-N protein in TG piglets only marginally extended their survival time by 3 days compared to wild-type piglets following challenge with HP-PRRSV. The results indicated that RNAi-based genetic modifications may could be utilized to breed virus-resistant livestock with stable siRNA expression and without siRNA-associated toxic complications. However, achieving complete resistance to the virus seems challenging.

## 6 Conclusions

To date, 8160 miRNAs have been identified in swine, with only a subset undergoing thorough characterization. This review presents a succinct survey of twenty-six miRNAs that influence PRRSV infection by regulating host factors or i binding to the viral genome. Among these miRNAs, miR-10a, miR-122, miR-218, ssc-miR-124a, miR-181, miR-506, miRNA let-7f-5p, miR-142-3p, miR-125b, miR-26a, miR-150, miR-331-3p, miR-210, miRNA let-7 family, miR-23, and miR-130b inhibit PRRSV infection, while miR-376b-3p, miR-373, miR-30c, miR-541-3p, miR-382-5p, miR-142-5p, miR-29, miR-296-3p, miR-22 and miR-24-3p promote PRRSV replication. It was demonstrated that miRNAs have been utilized in the development of highly effective live attenuated vaccines for various viruses (132–134). More importantly, intranasal delivery or intramuscular injection of miR-130b-3p, miRNA let-7, miR-181, and miR-331-3p can inhibit viral replication and alleviate PRRSV-induced lung injury *in vivo*, suggesting miRNAs mediated gene silence may be a promising strategy for controlling PRRSV infection. Despite significant progress in understanding the interplay between miRNAs and target genes associated with PRRSV infection, many miRNAs relevant to PRRSV are still in the early preclinical stages. Further investigations are imperative to develop innovative miRNA-based therapeutic drugs and overcome the challenges associated with current PRRS treatments. Clinical applications of miRNAs are emerging, with Miravirsen, an innovative anti-miRNA therapeutic, currently undergoing Phase 2b trials. This progress indicated that the potential feasibility of miRNA-based therapy. Viral vaccines engineered with miRNA target sequences are currently in the preclinical stage, further supporting the envisioned application of miRNA-based therapy for PRRSV infection in the future.

Additionally, the exact mechanism of initiating miRNA expressions following PRRSV infection is still unraveled. It has been observed that PRRSV infection can augment the activity of the miR-10a promoter by downregulating IRF8. In addition to transcription factors, the regulation of miRNA expressions may also involve crucial contributions from epigenetic modifications and lncRNA/circRNA. An exploration of the regulatory mechanisms underlying miRNA expressions in PRRSV-infected cells will help to understand the pathogenesis of PRRSV infection, and valuable insights into formulating strategies for the prevention and control of PRRSV infection.

## Author contributions

XH: Conceptualization, Funding acquisition, Investigation, Resources, Writing – original draft, Writing – review & editing. WL: Software, Visualization, Writing – review & editing.
